# Lytic Transglycosylase Deficiency Increases Susceptibility to β‐lactam Antibiotics But Reduces Susceptibility to Vancomycin in *Escherichia coli*

**DOI:** 10.1111/1348-0421.13227

**Published:** 2025-05-26

**Authors:** Takahiko Kimura, Kazuya Ishikawa, Ryosuke Nakagawa, Kazuyuki Furuta, Chikara Kaito

**Affiliations:** ^1^ Laboratory of Molecular Biology, Graduate School of Medicine, Dentistry, and Pharmaceutical Sciences Okayama University Okayama Japan

**Keywords:** *Escherichia coli*, lytic transglycosylase, seesaw effect, vancomycin, β‐lactam antibiotics

## Abstract

In *Staphylococcus aureus*, a gram‐positive pathogen, vancomycin‐resistant strains become susceptible to β‐lactam antibiotics, referred to as the “seesaw effect.” However, in gram‐negative bacteria, the phenomenon is less clear. Here, we analyzed the gene‐knockout effects of eight lytic transglycosylases (*slt, mltA, mltB, mltC, mltD, mltE, mltF, mltG*) on antibiotic sensitivity in *Escherichia coli*. Knockout of both *slt* and *mltG* increased sensitivity to β‐lactam antibiotics and reduced sensitivity to vancomycin. The β‐lactam antibiotic sensitivity and vancomycin resistance of the *slt*‐knockout mutant were abolished by the introduction of the wild‐type *slt* gene but remained unchanged by the introduction of the mutant *slt* gene encoding an amino acid substitution variant of the transglycosylase catalytic centre. The double‐knockout strain for *slt* and *mltB* was more sensitive to ampicillin and more resistant to vancomycin than each single‐knockout strain. The double‐knockout strain for *slt* and *mltG* was more sensitive to ampicillin and more resistant to vancomycin than each single‐knockout strain. These results suggest that loss of lytic transglycosylase activity causes β‐lactam antibiotic sensitivity and vancomycin resistance in *E. coli*.

**Abbreviations**
CTAB: hexadecyltrimethylammonium bromide
GlcNAc: N‐acetylglucosamine
LB: lysogeny broth
MurNAc: N‐acetylmuramic acid

## 1 Introduction

In Gram‐positive *Staphylococcus aureus* and *Bacillus subtilis*, strains that have become resistant to vancomycin, a glycopeptide antibiotic, become susceptible to β‐lactam antibiotics (seesaw effect). Methicillin‐resistant *Staphylococcus aureus* produces penicillin‐binding protein 2 A, a DD‐transpeptidase that is insensitive to β‐lactam antibiotics, and penicillin‐binding protein 4, which has DD‐transpeptidase, DD‐carboxypeptidase, and β‐lactamase activities. Decreased expression of these proteins in Methicillin‐resistant *Staphylococcus aureus* strains leads to vancomycin resistance by increasing the amount of d‐Ala‐d‐Ala, a target of vancomycin, and also causes susceptibility to β‐lactam antibiotics [1, 2, 3, 4]. Additionally, in *B. subtilis*, knockout of putative glycosyltransferase *ykcB* confers vancomycin resistance and susceptibility to β‐lactam antibiotics [5]. In contrast, Gram‐negative bacteria such as *Escherichia coli* are naturally resistant to vancomycin due to the presence of an outer membrane [6]; thus, a seesaw effect in *E. coli* has not been studied extensively. Recently, it was reported that in *E. coli*, a knockout of *dacA* encoding PBP5, a DD‐carboxypeptidase involved in cell wall synthesis, causes vancomycin resistance and β‐lactam antibiotic susceptibility (seesaw effect) [7]. But other *E. coli* genes involved in a seesaw effect are unclear.

*E. coli* has eight lytic transglycosylases: soluble Slt, outer membrane‐associated MltA, MltB, MltC, MltD, MltE, and MltF, and inner membrane‐spanning MltG [8, 9, 10, 11, 12, 13]. Although there are differences in exo‐ and endo‐degradation, all lytic transglycosylases cleave the β‐1,4 glycosidic bond between N‐acetylglucosamine (GlcNAc) and N‐acetylmuramic acid (MurNAc) in peptidoglycan, generating GlcNAc‐1,6‐anhydroMurNAc [13, 14]. It has been reported that *slt*‐ and *mltG*‐deficient strains are susceptible to β‐lactam antibiotics [15, 16], but a comprehensive analysis of the susceptibility of lytic transglycosylase gene‐deficient strains to β‐lactam antibiotics and vancomycin has not been conducted. In this study, we aimed to analyze the effect of the knockout of eight lytic transglycosylase genes in *E. coli* on β‐lactam antibiotic susceptibility and vancomycin resistance.

## 2 Materials and Methods

### 2.1 Bacterial Strains and Culture Conditions

*E. coli* KP7600 and the gene knockout strains were cultured on Lysogeny broth (LB) agar medium, and the colonies were aerobically cultured in LB liquid medium at 37°C. *E. coli* harbouring pMWCm were cultured in an LB liquid medium containing 25 μg/mL chloramphenicol. The bacterial strains used in this study are listed in Table 1.

**Table 1:** Bacterial strains and plasmids used in this study.

Strains or plasmid	Genotypes or characteristics	Source or reference
Strains		
KP7600	W3110 type‐A, F^−^, *lacI* ^q^, *lacZ*ΔM15, *galK2*, *galT22*, λ^−^, IN (*rrnD‐rrnE*)1	NBRP
JW4355	BW25113 Δ*slt*::*kan* Kan^r^	NBRP
JW4111	BW25113 Δ*ampC*::*kan* Kan^r^	NBRP
JW0627	BW25113 Δ*dacA*::*kan* Kan^r^	NBRP
JW2784	BW25113 Δ*mltA*::*kan* Kan^r^	NBRP
JW2671	BW25113 Δ*mltB*::*kan* Kan^r^	NBRP
JW5481	BW25113 Δ*mltC*::*kan* Kan^r^	NBRP
JW5018	BW25113 Δ*mltD*::*kan* Kan^r^	NBRP
JW5821	BW25113 Δ*mltE*::*kan* Kan^r^	NBRP
JW2542	BW25113 Δ*mltF*::*kan* Kan^r^	NBRP
JW1083	BW25113 Δ*mltG*::*kan* Kan^r^	NBRP
0001	KP7600 Δ*slt*::*kan* Kan^r^ (transduction from Keio collection JW4355)	This study
0002	KP7600 Δ*mltA*::*kan* Kan^r^ (transduction from Keio collection JW2784)	This study
0003	KP7600 Δ*mltB*::*kan* Kan^r^ (transduction from Keio collection JW2671)	This study
0004	KP7600 Δ*mltC*::*kan* Kan^r^ (transduction from Keio collection JW5481)	This study
0005	KP7600 Δ*mltD*::*kan* Kan^r^ (transduction from Keio collection JW5018)	This study
0006	KP7600 Δ*mltE*::*kan* Kan^r^ (transduction from Keio collection JW5821)	This study
0007	KP7600 Δ*mltF*::*kan* Kan^r^ (transduction from Keio collection JW2542)	This study
0008	KP7600 Δ*mltG*::*kan* Kan^r^ (transduction from Keio collection JW1083)	This study
0009	KP7600 Δ*ampC*::*kan* Kan^r^ (transduction from Keio collection JW4111)	This study
0010	KP7600 Δ*ampC* markerless	This study
0011	KP7600 Δ*ampC* markerless, Δ*slt*::*kan* Kan^r^	This study
0012	KP7600 Δ*dacA*::*kan* Kan^r^ (transduction from Keio collection JW0627)	This study
0013	KP7600 Δ*dacA* markerless	This study
0014	KP7600 Δ*dacA* markerless, Δ*slt*::*kan* Kan^r^	This study
0015	KP7600 Δ*slt* markerless	This study
0016	KP7600 Δ*mltB*::kan Kan^r^, Δ*slt* markerless	This study
0017	KP7600 Δ*mltG*::kan Kan^r^, Δ*slt* markerless	This study
JM109	Host strain for cloning	Takara Bio
Plasmids		
pMW118	A low copy plasmid, Amp^r^	Nippon Gene
pMWCm	A low copy plasmid, Cm^r^	This study
pslt	pMW118‐Cm^r^ with *slt*, Cm^r^	This study
pslt‐E505A	pMW118‐Cm^r^ with E505A *slt*, Cm^r^	This study
pCP20	FLP recombinase, Amp^r^	[17]

### 2.2 Genetic Manipulation

To obtain the *slt*, *mltA*, *mltB*, *mltC*, *mltD*, *mltE*, *mltF*, *mltG*, *ampC*, and *dacA* knockout strains, transduction with P1 *vir* was performed using gene knockout strains from the Keio Collection libraries as the phage donor and KP7600 as the recipient strain. To generate the *ampC* markerless knockout strain, the *ampC* knockout strain was transformed with pCP20 expressing FLP recombinase. After confirming the loss of the kanamycin cassette, the mutant was incubated at 43°C, and pCP20 was removed. The *slt*/*ampC* double knockout strain was generated by transduction with P1 *vir* using the *slt* knockout strain as the phage donor and the *ampC* markerless knockout strain as the recipient strain. To generate *dacA* markerless knockout and *slt* markerless knockout strains, *dacA* and *slt* knockout strains were transformed with pCP20 expressing FLP recombinase. After confirming the loss of the kanamycin cassette, the mutants were grown at 37°C, and pCP20 was removed. The *slt*/*dacA*, *slt*/*mltB*, and *slt*/*mltG* double knockout strains were created by transduction with P1 *vir* using the gene‐knockout strain as the phage donor and the markerless gene knockout strain as the recipient strain. To generate pMWCm, the DNA fragment of pMW118, excluding the Amp resistance marker, and the DNA fragment of chloramphenicol resistance marker of pKD3, were amplified by polymerase chain reaction (PCR), digested with XhoI and BglII, and ligated. To obtain a plasmid carrying *slt*, a DNA fragment containing the *slt* gene was amplified by PCR using the genomic DNA of KP7600 as a template and cloned into SphI and EcoRI sites of pMWCm. Amino acid substitution mutation (E505A) was introduced into the *slt* gene by thermal cycling reaction using primer pairs (E505A‐F and E505A‐R) and pMWCm‐slt as a template. The mutation was confirmed by DNA sequencing. The plasmids and oligonucleotide primers used in this study are listed in Tables 1 and 2.

**Table 2:** Primers used in this study.

Primer name	Sequence (5'–3')
pMW118_Del_F_XhoI	CTCCTCGAGAGCAAAAACAGGAAGGCAAA
pMW118_Del_R_BglII	AGAAGATCTCGTCAGACCCCGTAGAAAAG
pKD3_CAT_F_XhoI	CTCCTCGAGGCGCGCCTACCTGTGACGGAA
pKD3_CAT_R_BglII	AGAAGATCTAACTTCATTTAAATGGCGCGCCTT
slt‐F‐SphI	GCAGCATGCGTTCACCACCGGAGAGGTTA
slt‐R‐EcoRI	GAAGAATTCATGGAGATCGTTTTGGTAGGC
E505A‐F	TGGCGATTGCTCGTCAGGCGAGCGCCTGGAATCCG
E505A‐R	CGGATTCCAGGCGCTCGCCTGACGAGCAATCGCCA

### 2.3 Evaluation of Bacterial Resistance to Antimicrobial Substance

The autoclaved LB agar medium was mixed with antibiotics or surfactants and poured into round or square dishes. Overnight bacterial cultures were serially diluted 10‐fold with LB liquid medium in a 96‐well microplate, and 5 μL of the diluted bacterial solutions were spotted. The plates were incubated overnight at 37°C. Colonies were photographed using a digital camera and the number of colonies was counted according to a previously described method [18, 19].

### 2.4 Measurement of β‐lactamase Activity

Measurement of β‐lactamase activity was performed according to a previously described method [20]. Briefly, 100 µL of the overnight bacterial culture was inoculated into 10 mL of LB liquid medium and incubated at 37°C with shaking until OD600 = 1.0, cefoxitin was added at a final concentration 4 µg/mL, and the cultures were aerobically incubated at 37°C for 1 h. The bacterial cells were collected by centrifugation at 4°C, 12,000 × *g* for 15 min, washed once with 50 mM sodium phosphate buffer (pH 7.0), suspended in the same buffer, and sonicated. The sample was centrifuged at 12,000 × *g* for 15 min at 4°C, and the supernatant was obtained. The protein content was determined by the Bradford method. The total protein (final concentration 10 µg/mL) of the collected supernatant was mixed with nitrocefin (final concentration 50 µM) dissolved in 50 mM sodium phosphate buffer (pH 7.0), and hydrolysis of nitrocefin was determined by measuring the absorbance at 486 nm every 30 s for 10 min at room temperature. The activity was calculated using the molar absorption coefficient of hydrolyzed nitrocefin at 486 nm of 20,500 M^−^¹ cm^−^¹.

### 2.5 Statistical Analysis

All statistical analyses were performed using Prism software (version 10.4.0, GraphPad Software).

## 3 Results

### 3.1 Knockout of Lytic Transglycosylase Genes Leads to Sensitivity to Ampicillin and Resistance to Vancomycin

**Figure 1:**
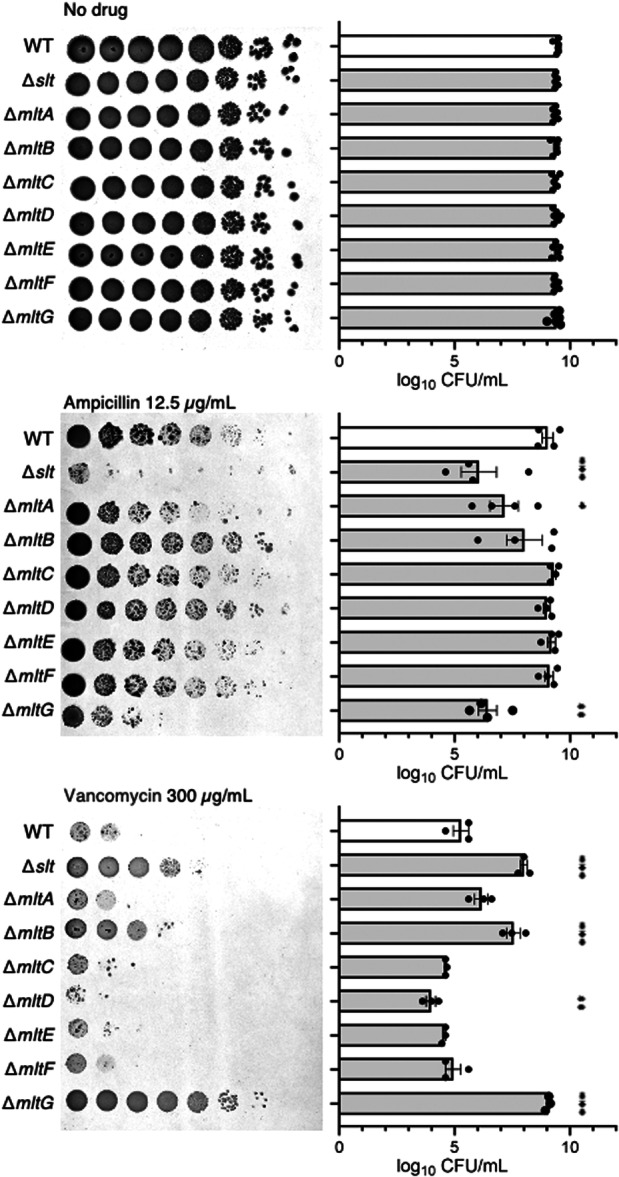
Knockout of lytic transglycosylases *slt* and *mltG* results in ampicillin sensitivity and vancomycin resistance. Overnight bacterial cultures of the wild‐type strain (WT) and gene‐knockout strains of *slt*, *mltA*, *mltB*, *mltC, mltD*, *mltE*, *mltF*, and *mltG* were serially diluted 10‐fold and spotted onto LB agar plates containing the indicated concentrations of antibiotics. The plates were incubated overnight at 37°C. Data shown are means ± standard error from at least three independent experiments, and statistical analysis was performed using one‐way analysis of variance with Dunnett's multiple comparisons test. *p < 0.05, **p < 0.01, ***p < 0.001.

We investigated whether gene knockouts of eight lytic transglycosylases (Slt, MltA, MltB, MltC, MltD, MltE, MltF, and MltG) of *E. coli* altered the susceptibility to ampicillin and vancomycin. The *slt*‐, *mltA*‐, and *mltG*‐knockout strains were susceptible to ampicillin (Figure 1). The *slt*‐, *mltB*‐, and *mltG*‐knockout strains were resistant to vancomycin, whereas the *mltD*‐knockout strain was weakly sensitive to vancomycin (Figure 1). These results suggest that the knockout of the lytic transglycosylase gene alters the sensitivity to ampicillin and vancomycin and that the *slt*‐ and *mltG*‐knockout strains show a seesaw effect of ampicillin sensitivity and vancomycin resistance.

### 3.2 *slt*‐Knockout Strain Shows Specific Alterations in Sensitivity to Cell Wall Synthesis Inhibitors

**Figure 2:**
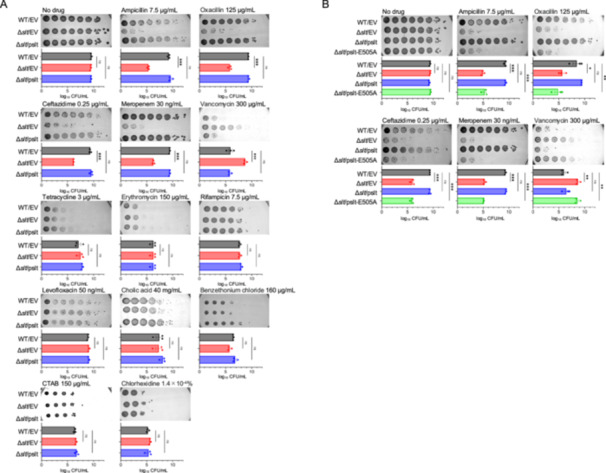
Knockout of *slt* leads to β‐lactam antibiotic sensitivity and vancomycin resistance but does not affect sensitivity to other antibiotics. (A) Overnight bacterial cultures of wild‐type strain transformed with an empty vector (WT/EV) and the *slt* knockout strain transformed with an empty vector (Δ*slt*/EV) or a plasmid carrying the *slt* gene (Δ*slt*/pslt) were serially diluted 10‐fold and spotted onto LB agar plates containing the indicated concentrations of drugs. The plates were incubated overnight at 37°C. The data represent the mean ± standard error from at least three independent experiments, and statistical analysis was performed using one‐way analysis of variance with Dunnett's multiple comparisons test. ***p < 0.001. (B) Overnight bacterial cultures of wild‐type strain transformed with an empty vector (WT/EV) and the *slt* knockout strain transformed with an empty vector (Δ*slt*/EV), a plasmid carrying the *slt* gene (Δ*slt*/pslt), or a plasmid carrying the mutated *slt* gene (Δ*slt*/pslt‐E505A) were serially diluted 10‐fold and spotted onto LB agar plates containing the indicated concentrations of antibiotics. The plates were incubated overnight at 37°C. The data represent the mean ± standard error from at least three independent experiments, and statistical analysis was performed using one‐way analysis of variance with Dunnett's multiple comparisons test. *p < 0.05, **p < 0.01, ***p < 0.001.

We investigated whether the *slt*‐knockout strain shows sensitivity to β‐lactam antibiotics other than ampicillin and alters susceptibility to antibiotics other than the cell wall synthesis inhibitors. The *slt*‐knockout strain was susceptible to the β‐lactam antibiotics oxacillin, ceftazidime, and meropenem compared to the wild‐type strain (Figure 2A). In contrast, the susceptibility to the protein synthesis inhibitors tetracycline and erythromycin, the RNA synthesis inhibitor rifampicin, the DNA replication inhibitor levofloxacin, the detergents cholic acid, benzalkonium chloride, and CTAB, and the disinfectant chlorhexidine did not differ between the wild‐type and *slt*‐knockout strains (Figure 2A). Introduction of a plasmid carrying the *slt* gene into the *slt*‐knockout strain abolished susceptibility to the β‐lactam antibiotics ampicillin, oxacillin, ceftazidime, and meropenem, and resistance to vancomycin (Figure 2A). These results suggest that *slt* gene knockout results in β‐lactam antibiotic susceptibility and vancomycin resistance without altering susceptibility to other antibiotics.

We investigated whether the susceptibility to β‐lactam antibiotics and vancomycin resistance of the *slt*‐knockout strain were due to the loss of Slt enzyme activity. Glutamate 505 of Slt is required for the initial step of β‐1,4 cleavage of the GlcNAc‐MurNAc bond of peptidoglycan [21, 22]. The introduction of wild‐type *slt* abolished the change in sensitivity to cell wall synthesis inhibitors caused by the *slt* knockout. In contrast, the introduction of slt‐E505A, where glutamic acid 505 in the active centre of Slt was replaced by alanine, did not affect the change in sensitivity to cell wall synthesis inhibitors caused by the *slt* knockout (Figure 2B). These results suggest that loss of Slt transglycosylase activity leads to susceptibility to β‐lactam antibiotics and resistance to vancomycin.

### 3.3 Endogenous β‐Lactamase *ampC* is not Involved in β‐Lactam Antibiotic Susceptibility and Vancomycin Resistance in the *slt*‐Knockout Strain

**Figure 3:**
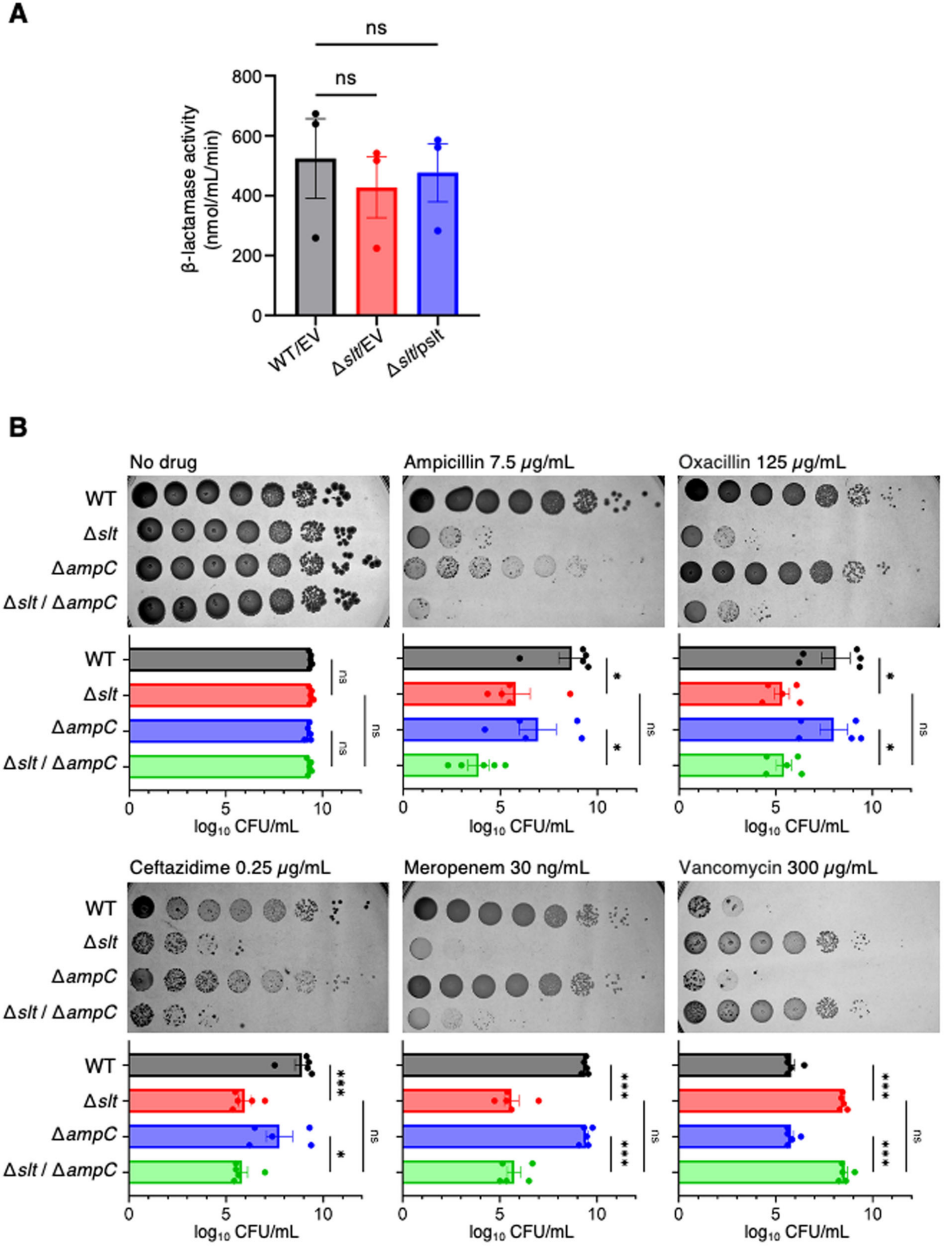
Endogenous β‐lactamase *ampC* does not contribute to β‐lactam antibiotic sensitivity in the *slt* knockout strain. (A) β‐lactamase activities of wild‐type strain transformed with empty vector (WT/EV), the *slt* knockout strain transformed with empty vector (Δ*slt*/EV), and the *slt* knockout strain transformed with a plasmid carrying the *slt* gene (Δ*slt*/pslt). The data depicts the mean ± standard error from three independent experiments, and statistical analysis was performed using one‐way analysis of variance with Dunnett's multiple comparisons test. (B) Overnight bacterial cultures of the wild‐type strain (WT), the *slt* knockout strain (Δ*slt*), the *ampC* knockout strain (Δ*ampC*), and the *slt*/*ampC* double knockout strain (Δ*slt*/Δ*ampC*) were serially diluted 10‐fold and spotted onto Lysogeny broth (LB) agar plates containing the indicated concentrations of antibiotics. The plates were incubated overnight at 37°C. The data represents the mean ± standard error from five independent experiments. Statistical analyses were performed using Šídák's multiple comparisons test. *p < 0.05, ***p < 0.001.

Gram‐negative bacteria, including *E. coli*, have endogenous β‐lactamases located on their chromosomes [23, 24]. Considering the possibility that the susceptibility of the *slt*‐knockout strain to β‐lactam antibiotics is due to a decrease in endogenous β‐lactamase activity, we measured the β‐lactamase activity of the *slt*‐knockout strain. There was no difference in β‐lactamase activity among the wild‐type, *slt*‐knockout, and complemented strains (Figure 3). We next examined whether the endogenous β‐lactamase *ampC* contributes to the β‐lactam antibiotic susceptibility of *slt*‐knockout strain. The *slt*/*ampC* double‐knockout strain was more susceptible to β‐lactam antibiotics than the *ampC* single‐knockout strain (Figure 3). Furthermore, the *slt*/*ampC* double‐knockout strain exhibited vancomycin resistance compared with the *ampC* single‐knockout strain, and the resistance level was similar to the *slt* single‐knockout strain (Figure 3). These results suggest that the endogenous β‐lactamase *ampC* is not involved in the β‐lactam antibiotic susceptibility and vancomycin resistance of the *slt*‐knockout strain.

### 3.4 *slt* Knockout Causes Vancomycin Resistance in a DD‐Carboxypeptidase *dacA*‐Dependent Manner

**Figure 4:**
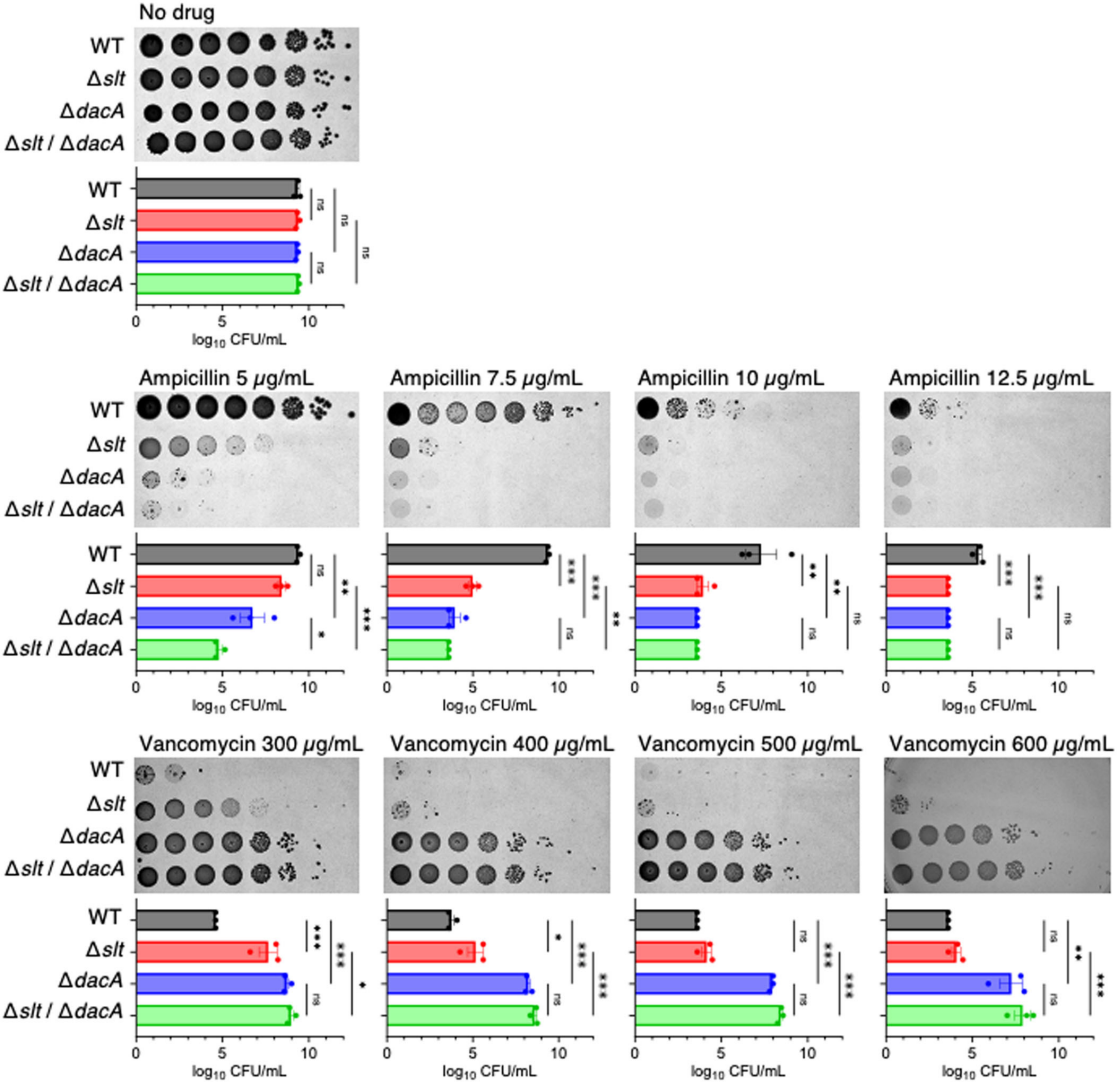
DD‐carboxypeptidase *dacA* is required for vancomycin resistance caused by the *slt* knockout. Overnight bacterial cultures of the wild‐type strain (WT), the *slt* knockout strain (Δ*slt*), the *dacA* knockout strain (Δ*dacA*), and the *slt*/*dacA* double knockout strain (Δ*slt*/Δ*dacA*) were serially diluted 10‐fold and spotted onto LB agar plates containing the indicated concentrations of antibiotics. The plates were incubated overnight at 37°C. Data are the mean ± standard error from three independent experiments. Statistical analyses were performed using Šídák's multiple comparisons test. *< 0.05, **p < 0.01, ***p < 0.001.

In *Escherichia coli*, Slt interacts with PBP3 and PBP7/8, while in *Pseudomonas aeruginosa*, Slt interacts with PBP1a, PBP1b, PBP5, and PBP7 [25, 26]. A knockout mutant of *dacA*, which encodes DD‐carboxypeptidase (PBP5), is sensitive to β‐lactam antibiotics and resistant to vancomycin [7]. Therefore, we hypothesised that Slt may contribute to changes in susceptibility to β‐lactam antibiotics and vancomycin by interacting with PBPs and investigated whether *dacA* (PBP5) is involved in the susceptibility to β‐lactam antibiotics and vancomycin resistance of the *slt*‐knockout strain. The *slt*/*dacA* double‐knockout strain was more sensitive to 5 µg/mL ampicillin than the *dacA* single‐knockout and *slt* single‐knockout strains (Figure 4). The *slt*/*dacA* double‐knockout strain showed resistance to 300–600 µg/mL vancomycin at the same level as the *dacA* single‐knockout strain (Figure 4). These results suggest that the *slt* knockout causes β‐lactam antibiotic susceptibility in a *dacA*‐independent manner, whereas the *slt* knockout causes vancomycin resistance in a *dacA*‐dependent manner.

### 3.5 Double Knockout of Lytic Transglycosylases Increases Susceptibility to β‐Lactam Antibiotics and Vancomycin Resistance

**Figure 5:**
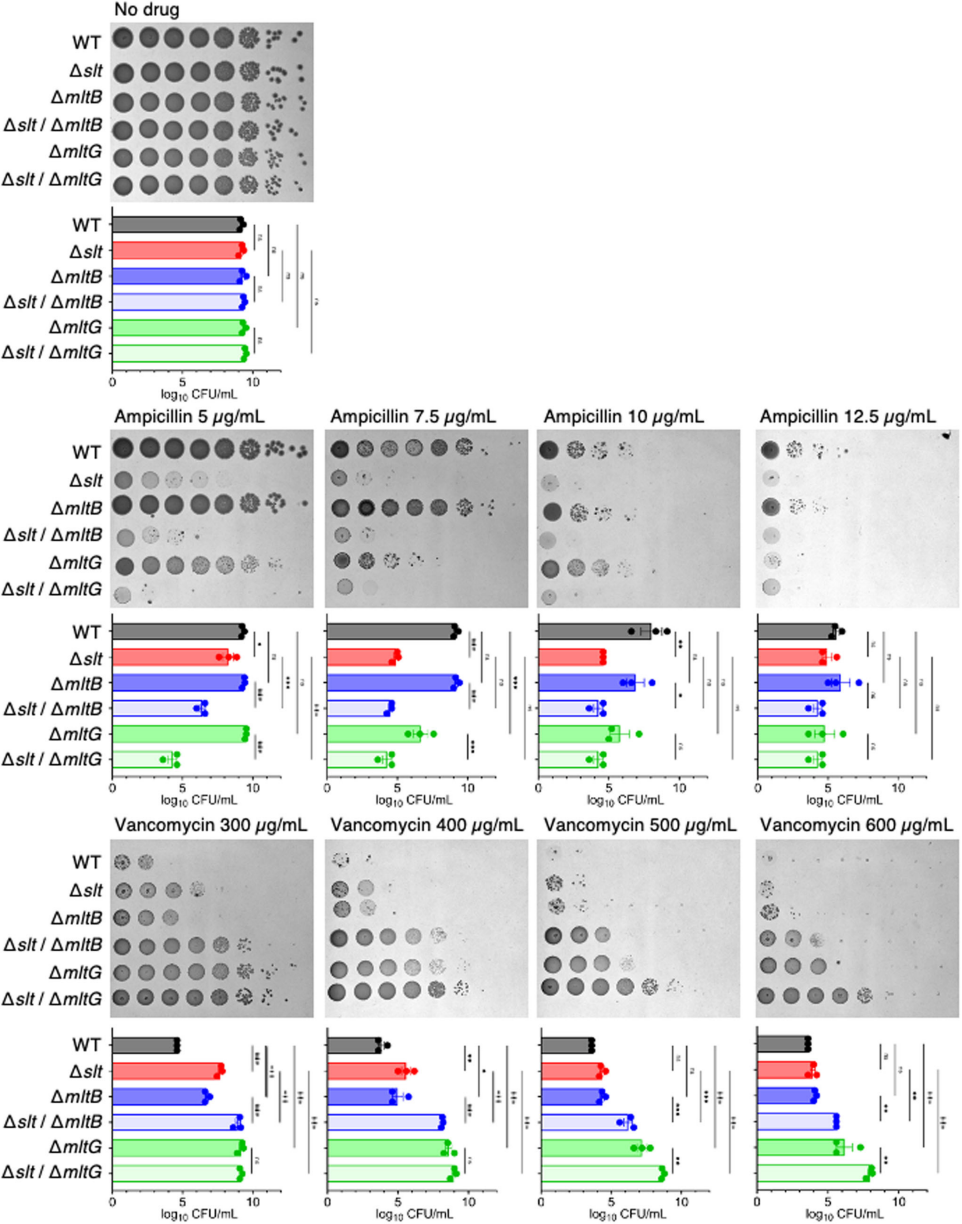
Double knockout of different lytic transglycosylases enhances β‐lactam antibiotic sensitivity and vancomycin resistance. Overnight bacterial cultures of the wild‐type strain (WT), the *slt* knockout strain (Δ*slt*), the *mltB* knockout strain (Δ*mltB*), the *slt*/*mltB* double knockout strain (Δ*slt*/Δ*mltB*), the *mltG* knockout strain (Δ*mltG*), and the *slt*/*mltG* double knockout strain (Δ*slt*/Δ*mltG*) were serially diluted 10‐fold and spotted onto LB agar plates containing the indicated concentrations of antibiotics. The plates were incubated overnight at 37°C. Data are the mean ± standard error from three independent experiments. Statistical analyses were performed using Šídák's multiple comparisons test. **p < 0.01, ***p < 0.001.

Based on the result that knockouts of *slt* and *mltG* led to ampicillin susceptibility and knockouts of *slt*, *mltB*, and *mltG* led to vancomycin resistance, we hypothesized that knockouts in multiple lytic transglycosylases cooperatively cause β‐lactam antibiotic susceptibility and vancomycin resistance. We constructed *slt*/*mltB* and *slt*/*mltG* double‐knockout strains and examined their ampicillin susceptibility and vancomycin resistance. The *slt*/*mltB* double‐knockout strain increased sensitivity to 5 µg/mL ampicillin compared with the *slt*‐knockout and *mltB*‐knockout strains, but showed enhanced resistance against 300–600 µg/mL vancomycin compared with the *slt*‐knockout and *mltB*‐knockout strains (Figure 5). The *slt*/*mltG* double‐knockout strain increased sensitivity to 5 µg/mL ampicillin compared with the *slt*‐knockout and *mltG*‐knockout strains and showed enhanced resistance against 500–600 µg/mL vancomycin compared with the *slt*‐knockout and *mltG*‐knockout strains (Figure 5). These results suggest that knockout of *slt* and *mltB* or that of *slt* and *mltG* additively confer ampicillin susceptibility and vancomycin resistance.

## 4 Discussion

This study revealed that knockout of the *slt* and *mltG* genes, among the eight lytic transglycosylase genes of *E. coli*, results in a seesaw effect of β‐lactam antibiotic susceptibility and vancomycin resistance. Furthermore, we demonstrated that double knockout of *slt* and *mltB* or that of *slt* and *mltG* enhances ampicillin susceptibility and vancomycin resistance. Furthermore, an *slt* gene carrying an amino acid substitution mutation that abolished the enzymatic activity of Slt lost the ability to revert the change in antibiotic sensitivity in the *slt*‐knockout strain to the wild‐type level. To our knowledge, this study is the first to demonstrate that loss of lytic transglycosylase activity in *E. coli* leads to β‐lactam antibiotic susceptibility and vancomycin resistance.

The results of the *slt*/*ampC* double knockout strain revealed that β‐lactam antibiotic susceptibility and vancomycin resistance caused by the *slt* deficiency are not mediated by endogenous β‐lactamase *ampC*. It has been proposed that Slt contributes to the defence against β‐lactam antibiotics by cleaving uncrosslinked glycan chains generated by exposure to β‐lactam antibiotics, thereby preventing the accumulation of abnormal peptidoglycan [27]. Thus, loss of lytic transglycosylase may confer susceptibility to beta‐lactam antibiotics by promoting the disruption of the peptidoglycan layer through the incorporation of uncrosslinked glycan chains upon exposure to these antibiotics.

Gram‐negative bacteria such as *E. coli* have a natural resistance to high molecular weight (molecular weight ≥ 600 Da) antibiotics such as vancomycin and erythromycin due to the presence of their outer membrane [6]. Some findings exist on the vancomycin resistance mechanism other than the mechanism involving the integrity of the outer membrane. When a point mutation in WaaL, an O‐antigen ligase of *E. coli*, causes lipopolysaccharide‐modified with lipid II‐derived moieties containing d‐Ala‐d‐Ala to be exposed on the outside of the outer membrane, vancomycin is trapped in the outer membrane, resulting in vancomycin resistance [28]. A deficiency of *E. coli* DD‐carboxypeptidase *dacA* (PBP5) leads to vancomycin resistance due to an increase in d‐Ala‐d‐Ala [7]. Slt interacts with PBPs in *E. coli* and *P. aeruginosa* [25, 26]. This study revealed that the *slt*‐knockout causes vancomycin resistance in a *dacA*‐dependent manner (Figure 4). This study also revealed that vancomycin resistance in the *slt*‐knockout strain was associated with the loss of Slt enzyme activity (Figure 2B). Therefore, it is possible that in strains lacking the lytic transglycosylases *slt* and *mltG*, loss of transglycosylase activity or loss of interaction between transglycosylase and PBPs affects PBP activity, resulting in vancomycin resistance due to an increase in the amount of d‐Ala‐d‐Ala. Further analyses are required to examine the changes in PBP activity and the amount of d‐Ala‐d‐Ala in *slt* and *mltG* knockout strains.

In this study, we demonstrated that double knockouts of either *slt*/*mltB* or *slt*/*mltG* exhibited enhanced sensitivity to β‐lactam antibiotics compared to single knockout strains of *slt*, *mltB*, and *mltG* (Figure 5). These findings suggest that a common inhibitor against Slt, MltB, and MltG could function as an effective adjuvant to potentiate β‐lactam efficacy. However, our data indicate that such antimicrobial adjuvants induce resistance of *E. coli* against 300 μg/mL vancomycin through inhibition of Slt, MltB, and MltG (Figure 5). Clinically, this vancomycin resistance mechanism is unlikely to pose significant concerns, as vancomycin is administered at serum concentrations of 15–20 μg/mL and is not typically used for *E. coli* infections [29]. In addition, inhibitors against lytic transglycosylases could have broader therapeutic potentials beyond the antibiotic potentiation. In *Acinetobacter baumannii*, *mltB* knockout increases susceptibility to host humoral immune factors such as complement, antimicrobial peptides, and oxidative stress, resulting in reduced bacterial loads in murine infection models [30]. In *Francisella novicida*, the *slt*‐knockout mutant shows impaired growth under acidic conditions, decreased bacterial viable cells in host monocytic cells, and attenuated virulence in mouse models [31, 32]. In *Burkholderia pseudomallei*, the *ltgG*‐knockout mutant exhibits reduced motility and virulence in murine infection model [33]. These findings suggest that inhibitors against lytic transglycosylases could be novel anti‐infection drugs capable of increasing bacterial sensitivity to β‐lactam as well as reducing bacterial virulence.

In conclusion, this study revealed the relationship between lytic transglycosylase and the seesaw effect in *E. coli*, which is important for elucidating the molecular mechanism of the seesaw effect common to both Gram‐positive and Gram‐negative bacteria.

## Conflicts of Interest

Chikara Kaito is the Editor‐in‐Chief of Microbiology and Immunology and a coauthor of this article. They were excluded from editorial decision‐making related to the acceptance and publication of this article.

## Data Availability

The authors have nothing to report.
